# Influence of KIR genes and their HLA ligands in the pathogenesis of leprosy in a hyperendemic population of Rondonópolis, Southern Brazil

**DOI:** 10.1186/1471-2334-14-438

**Published:** 2014-08-12

**Authors:** Luciana Ribeiro Jarduli, Hugo Vicentin Alves, Fabiana Covolo de Souza-Santana, Elaine Valim Camarinha Marcos, Ana Carla Pereira, Ida Maria Foschiani Dias-Baptista, Vinícius Medeiros Fava, Marcelo Távora Mira, Milton Ozório Moraes, Marcos da Cunha Lopes Virmond, Jeane Eliete Laguila Visentainer

**Affiliations:** Laboratório de Imunogenética, Departamento de Ciências Básicas da Saúde, Universidade Estadual de Maringá, Av. Colombo, 5790 Maringá, PR CEP 87020-900, Brazil; Instituto Lauro de Souza Lima, Bauru, SP Brazil; Pontifícia Universidade Católica do Paraná, Curitiba, PR Brazil; Instituto Oswaldo Cruz, FIOCRUZ, Rio de Janeiro, RJ Brazil

**Keywords:** Leprosy, KIR genes, NK cells

## Abstract

**Background:**

The objective of this study was to investigate the association between KIR genes and the immunopathogenesis of leprosy.

**Methods:**

The types of KIR and HLA genes were evaluated by PCR-SSOP-Luminex in 408 patients with leprosy and 413 healthy individuals. Statistical analysis was performed using the Chi-square or Fisher’s exact test and stepwise multivariate analysis.

**Results:**

There was a higher frequency of activating KIR genes (*KIR2DS1*, *2DS2* and *3DS1*) together with their HLA ligands in the tuberculoid (TT) group as compared to the lepromatous leprosy (LL) group. *KIR2DL2/2DL2-C1* was more frequent in the patient, TT and LL groups than in the control group. Borderline patients presented a higher frequency of inhibitory pairs when compared to the control group, and a higher frequency of activating pairs as compared to the LL group. Multivariate analysis confirmed the associations and demonstrated that being a female is a protective factor against the development of the disease *per se* and the more severe clinical form.

**Conclusions:**

This study showed that activating and inhibitory KIR genes may influence the development of leprosy – in particular, activating genes may protect against the more aggressive form of the disease – thereby demonstrating the role of NK cells in the immunopathology of the disease.

**Electronic supplementary material:**

The online version of this article (doi:10.1186/1471-2334-14-438) contains supplementary material, which is available to authorized users.

## Background

Leprosy or Hansen’s disease is a chronic, slowly developing infectious disease caused by *Mycobacterium leprae*, which global prevalence has been decreasing over recent years due to effective multiple drug therapy. However, the detection of new cases remains constant at approximately 250,000 per year, with pockets of high endemicity existing in countries such as Brazil, Angola, Africa, India, Nepal, and the United Republic of Tanzania [1]. In Brazil, leprosy has been diagnosed in all regions of the country, with the Northern and Mid-Western regions being hyperendemic and the Northeastern region having high endemicity [2].

The most widely used classification model for the disease is that of Ridley-Jopling, which divides the disease into five polar and interpolar forms: Tuberculoid (TT), Borderline-Tuberculoid (BT), Borderline-Borderline (BB), Borderline-lepromatous leprosy (BL) and Lepromatous leprosy (LL) [3]. Initially, the disease is of indeterminate form, which may regress spontaneously or evolve into one of these stages. The clinical manifestations of the disease depend on factors such as the ability of the bacilli to proliferate and the immune response presented by the host [4].

Over the years, research has been aimed at better understanding the genetics of susceptibility to the different clinical forms of leprosy. The clinical and pathological spectrum of leprosy and the epidemiological, geographical, and ethnic heterogeneity might be explained by genetic differences in the host’s resistance. While some loci affect the intrinsic susceptibility to leprosy itself, others modify the clinical form of the disease [5]. Among the candidate genes for an individual’s susceptibility or resistance to leprosy are the KIR (Killer Immunoglobulin-Like Receptor) genes. These genes span over approximately 150 Kb of the LCR (Leukocyte Receptor Complex) region of chromosome 19q13.4, which encodes KIRs, members of a group of regulatory molecules present on the surface of NK (Natural Killer) cells [6].

The KIRs are the main functional regulators of NK cells; the activity of these effector cells is determined by the balance between their activation and inhibition that occurs as a result of the binding of KIRs with HLA class I molecules present in all nucleated cells [7]. Most KIRs bind to HLA-C molecules; the KIR2DL2 and KIR2DL3 inhibitory receptors interact with HLA-C of Group 1, while KIR2DL1 recognizes HLA-C of Group 2. In the absence of these inhibitory receptors, NK cells can be activated by activating receptors, such as KIR2DS1 and KIR2DS2 [8]. The genotype may predispose a subject to certain interactions that vary from activation to inhibition depending on the individual’s KIR/HLA profile [9].

To date, only one study has reported an association between KIR genes and their HLA ligands with leprosy phenotypes, using a population sample of 165 patients from south Brazil [10]. Thus, the aim of this study was to investigate the association between KIR genes and their HLA ligands in the development of leprosy and its clinical forms in patients from the hyperendemic region of Rondonópolis, Mato Grosso, Mid-Western Brazil, ultimately aiming to better understand the immunopathogenic mechanisms of *M. leprae*.

## Methods

### Population and epidemiological data collection

This study was approved by the Research Ethics Committee of the Instituto Lauro de Souza Lima Bauru, São Paulo, Brazil, and funded by the Conselho Nacional de Desenvolvimento Científico e Tecnológico (CNPq). All participants signed an informed consent form agreeing to participate in the study. Data for epidemiological characterization were collected in government healthcare clinics by applying a questionnaire and by revisiting medical records and notification registration forms of patients treated between 2006 and 2009.

This case–control study was conducted using a population sample from Rondonópolis (Mid-Western State of Mato Grosso, Brazil: latitude 16°28′15″ South and longitude 54°38′08″ West), consisting of 408 patients with leprosy (250 men and 158 women) with a median age of 41 years treated in government healthcare clinics of the municipality of Rondonópolis. The patients were classified into distinct groups according to the Ridley-Jopling criteria [3]: LL (5.14%), borderline (BL 15.4%; BB 25.8%; BT 39.4%) and TT (14.7%), with 25 patients (6.12%) presenting the indeterminate form of the disease. The control group consisted of healthy individuals 413 (249 men and 164 women) with a median age of 42 years. The following criteria were used while forming the control group: no family and/or personal history with leprosy and no history of chronic infections, inflammation, or autoimmune diseases. The control group was matched to the case group according to epidemiological characteristics such as ethnicity, gender, age and geographical region.

### DNA extraction

After collecting 5 mL of peripheral venous blood in EDTA anticoagulant, the extraction and purification of DNA were performed by selective precipitation using the technique described by John et al. [11].

### Typing of the KIR genes and HLA class I alleles

Typing of the KIR genes and HLA class I alleles (HLA-A, -B, -C) was performed by PCR-SSOP (Polymerase Chain Reaction-Sequence Specific Oligonucleotide Probes) using the SSO LabType® kit (One Lambda, San Diego, CA, USA) after evaluating the purity and adjusting the concentration of the DNA (20 ng). The amplified product was hybridized with microspheres bound to probes specific for the KIR gene and HLA class I alleles. The resulting products were analysed using a LUMINEX® flow cytometer and the results were compared by HLA Fusion™ (One Lambda, San Diego, CA, USA).

### Statistical analysis

The frequencies of KIR genes and HLA class I alleles were obtained by direct counting and by comparing these frequencies between patients and controls using the Chi-square test with Yates correction or Fisher’s exact test using a 2 × 2 contingency table with a 95% confidence interval (95% CI). The risk of developing leprosy and its clinical forms was calculated by determining the OR (odds ratio) for *P-*values < 0.05 according to Wolf [12], using the statistics program OpenEpi v. 2.3.1 (http://www.openepi.com/OE2.3/Menu/OpenEpiMenu.htm). *P* values were corrected by the Bonferroni inequality method, by multiplying them by the number of KIR genes analysed.

Subsequently, multivariate logistic regression analysis was performed using stepwise method of data analysis, using age, gender and ethnicity as covariates, employing IBM® SPSS® 20.0 Statistics software (SPSS, IBM Inc., Chicago, IL, USA) with the level of significance set at 5% (*P-*value ≤ 0.05).

## Results

The characteristics of the study population with respect to age, gender and ethnic group are shown in Table 1. Both the mean and median ages were similar among all groups (Total Patients, LL, Borderline, TT and Control), demonstrating a good match between cases and controls.

**Table 1:** **Distribution of the population of patients and healthy individuals according to clinical forms of leprosy, age, gender and ethnic group**

		*Per se*	LL	Borderline	TT	Controls
		N = 408	N = 21	N = 302	N = 60	N = 413
Age	(Mean ± SD)	42.0 ± 16.4	45.7 ± 15.2	42.8 ± 15.9	37.4 ± 16.8	42.0 ± 14.5
	(Median)	41	45	42	37	42
		n (%)	n (%)	n (%)	n (%)	n (%)
Gender	Male	250 (61.2)	17 (80.9)	194 (64.2)	25 (41.6)	249 (60.2)
	Female	158 (38.7)	4 (19.0)	108 (35.7)	35 (58.3)	164 (37.7)
Ethnic	Caucasian	146 (35.7)	8 (38.0)	115 (38.0)	14 (23.3)	137 (28.3)
Group	Black	16 (3.92)	0 (0.0)	11 ( 3.64)	3 ( 5.0)	17 ( 4.11)
	Afro-Brazilian	246 (60.2)	13 (61.9)	176 (58.2)	43 (71.6)	259 (62.7)

**NOTE:** Distribution of patients classified according to criteria of Ridley & Joplin: Leprosy (*per se*), Tuberculoid (TT), Borderline (BL, BB, BT), and Lepromatous (LL); according to age, gender (male and female) and ethnicity (Caucasian, Black and Afro-Brazilian). Values in parentheses correspond to the percentages of each variable.

There was a predominance of men in the total patients (61.2%), LL (80.9%), Borderline (64.2%) and Control groups (60.2%), while the TT group was composed predominantly of females (58.3%). There was a predominance of Afro-Brazilians followed by Caucasians and Blacks in all groups, with similar proportions of ethnicity in each group.

The distribution of KIR gene frequencies is shown in Table 2. There were significant differences in the allelic distributions of the *KIR2DL1* gene when comparing the Total Patient and Control Groups (87.0% vs. 96.2%, *P-*value < 0.001, *Pc* < 0.014, OR = 0.3, 95% CI = 0.2-0.5) and Borderline and Control Groups (86.0% vs. 96.2%, *P-*value < 0.001, *Pc* < 0.014, OR = 0.2, 95% CI: 0.1-0.5). There was also a trend (*P-*value = 0.07) for positive association between the *KIR3DS1* activating gene and the TT when compared to the LL form of the disease (43.3% vs. 19.0%).

**Table 2:** **Distribution of the frequencies of KIR genes in patients with leprosy and their clinical forms and healthy individuals in the population of Rondonopolis, MT**

Genes	*Per se*	LL	Borderline	TT	Controls
	N = 408	N = 21	N = 302	N = 60	N = 413
	n (%)	n (%)	n (%)	n (%)	n (%)
*KIR2DL1*	355 (87.0)^a^	19 (90.4)	260 (86.0)^a^	56 (93.3)	397 (96.2)^a^
*KIR2DL2*	290 (72.0)	14 (66.6)	209 (69.2)	48 (80.0)	300 (72.6)
*KIR2DL3*	363 (88.9)	18 (85.7)	272 (90.0)	53 (88.3)	380 (92.0)
*KIR2DL4*	405 (99.2)	21 (100)	300 (99.3)	60 (100)	409 (99.0)
*KIR2DL5*	227 (55.6)	8 (38.0)	167 (55.2)	36 (60.0)	225 (54.4)
*KIR3DL1*	388 (95.0)	21 (100)	286 (94.7)	58 (96.6)	392 (94.9)
*KIR3DL2*	405 (99.2)	21 (100)	301 (99.6)	59 (98.3)	413 (100)
*KIR3DL3*	406 (99.5)	21 (100)	301 (99.6)	60 (100)	413 (100)
*KIR2DS1*	151 (37.0)	5 (23.8)	112 (37.0)	25 (41.6)	156 (37.7)
*KIR2DS2*	215 (52.6)	10 (47.6)	159 (52.6)	30 (50.0)	197 (47.6)
*KIR2DS3*	123 (30.1)	4 (19.0)	90 (29.8)	19 (31.6)	129 (60.5)
*KIR2DS4*	387 (94.8)	21 (100)	286 (94.7)	57 (95.0)	386 (93.4)
*KIR2DS5*	144 (35.2)	5 (23.8)	107 (35.4)	23 (38.3)	157 (38.0)
*KIR3DS1*	148 (36.2)	4 (19.0)^b^	107 (35.4)	26 (43.3)^b^	153 (37.0)

**NOTE:** Data represent the number of KIR genes and values in parentheses correspond to the percentages of each gene. TT: Tuberculoid, Borderline (BL, BB, BT), LL: Lepromatous.

^a^ *KIR2DL*1: *P* < 0.001, *Pc <* 0.014, OR = 0.26 (0.15 – 0.48) to *per se vs*. controls *P* < 0.001, Pc < 0.014, OR = 0.24 (0.13 – 0.45) to Borderline *vs*. controls.

^b^ *KIR3DS1*: *P* = 0.07 (tendency) to TT *vs*. LL.

Data on the distribution of KIR genes with their correlated HLA class I ligands are shown in Table 3. For the *KIR2DS2-C1* activating receptor, the frequency was lower for the Control Group (20.5%) as compared to the Total Patient Group (27.2%: *P-*value = 0.031; OR = 1.4; 95% CI: 1.0-2.0) and the TT Group (33.3%: *P-*value = 0.045; OR = 1.9; 95% CI = 1.1-3.5). For the inhibitory genes, there was a higher frequency of the homozygous *KIR2DL2* receptor in the presence of *C1* (*KIR2DL2*/*2DL2-C1*) in the Total Patient Group when compared to the Control Group (5.4% vs. 2.17%; *P-*value = 0.024; OR = 2.6; 95% CI = 1.2-5.6) and higher frequencies for the TT (8.3% vs. 2.17%; *P-*value = 0.045; OR = 4.1; 95% CI = 1.3-12.6) and LL (14.2% vs. 2.17%; *P-*value = 0.032; OR = 7.5; 95% CI = 1.9-30.1) clinical forms compared to the Control Group. In the analysis of the *KIR2DL2*/*2DL3* haplotype with its homozygous C1 ligand (*KIR2DL2*/*2DL3* - *C1*/*C1*), the frequency was lower in the Total Patient Group than the Control Group (13.7% vs. 19.8%; *P-*value = 0.023; OR = 0.6; 95% CI: 0.4-0.9).

**Table 3:** **Frequencies of KIR genes in the presence of their ligands HLA Class I in patients with leprosy and their clinical forms and healthy individuals in the population of Rondonópolis, MT**

*KIR – HLA Class I*	*Per se*	LL	Borderline	TT	Controls
	N = 408	N = 21	N = 302	N = 60	N = 413
	n (%)	n (%)	n (%)	n (%)	n (%)
*KIR2DL1 – C2*	173 (42.4)	10 (47.6)	121 (40.0)	29 (48.3)	188 (45.5)
*KIR2DL1 – C2/C2*	82 (20.0)	5 (23.8)	59 (19.5)	14 (23.3)	79 (19.1)
*KIR2DL2 – C1*	145 (35.5)	8 (38.0)	95 (31.4)	27 (45.0)	134 (32.4)
*KIR2DL2 – C1/C1*	71 (17.4)	2 (9.52)	56 (18.5)	11 (18.3)	95 (23.0)
*KIR2DL3 – C1*	174 (42.6)	9 (42.8)	128 (42.3)	26 (43.3)	184 (44.5)
*KIR2DL3 – C1/C1*	92 (22.5)	3 (14.2)	71 (23.5)	13 (21.6)	116 (28.0)
*KIR3DL1 – Bw4*	153 (37.5)	7 (33.3)	118 (39.0)	18 (30.0)	164 (39.7)
*KIR3DL1 – Bw4/Bw4*	44 (10.7)	2 (9.52)	33 (10.9)	7 (11.6)	54 (13.0)
KIR3DL2 – A*03/*11	99 (24.2)	4 (19.0)	74 (24.5)	15 (25.0)	86 (20.8)
*KIR2DS1 – C2*	70 (17.1)	2 (9.52)	48 (15.8)	14 (23.3)	74 (17.9)
*KIR2DS1 – C2/C2*	38 (9.31)	2 (9.52)	27 (8.94)	7 (11.6)	25 (6.05)
*KIR2DS2 – C1*	111 (27.2)^a^	5 (23.8)	74 (24.5)	20 (33.3)^a^	85 (20.5)^a^
*KIR2DS2 – C1/C1*	51 (12.5)	3 (14.2)	39 (12.9)	6 (10.0)	60 (14.5)
*KIR3DS1 – Bw4*	57 (13.9)	1 (4.76)	46 (15.2)	7 (11.6)	59 (14.2)
*KIR3DS1 – Bw4/Bw4*	21 (5.14)	1 (4.76)	16 (5.29)	4 (6.66)	23 (5.56)
*KIR2DL2/2DL2 – C1*	22 (5.39)^b^	3 (14.2)^b^	10 (3.31)^b^	5 (8.33)^b^	9 (2.17)^b^
*KIR2DL2/2DL2 – C1/C1*	14 (3.43)	0 (0.0)	11 (3.64)	2 (3.33)	10 (2.42)
*KIR2DL2/2DL3 – C1*	122 (29.9)	6 (28.5)	88 (29.1)	19 (31.6)	124 (30.0)
*KIR2DL2/2DL3 – C1/C1*	56 (13.7)^c^	2 (9.52)	43 (14.2)	11 (18.3)	82 (19.8)^c^
*KIR2DL3/2DL3 – C1*	55 (13.4)	4 (19.0)	44 (14.5)	5 (8.33)	62 (15.0)
*KIR2DL3/2DL3 – C1/C1*	34 (8.33)	1 (4.76)	25 (8.27)	4 (6.66)	32 (7.74)

**NOTA:** Data represent the number of *KIR* genes and their respective ligands HLA Class I; the values in parentheses correspond to the percentages of each pair KIR-HLA.

TT: Tuberculoid, Borderline (BL, BB, BT), LL: Lepromatous.

^a^ *KIR2DS2 – C1*: *P =* 0.031, OR = 1.4 (1.0 – 2.0) to *per se vs*. controls; *P =* 0.045, OR = 1.9 (1.1 – 3.5) to TT *vs*. controls.

^b^ *KIR2DL2/2DL2 – C1: P =* 0.024, OR = 2.6 (1.2 – 5.6) to *per se vs*. controls; *P =* 0.045, OR = 4.1 (1.3 – 12.6) to TT *vs*. controls; *P =* 0.032, OR = 7.5 (1.9 – 30.1) to LL *vs*. controls; *P =* 0.057 (tendency) to Borderline *vs*. LL.

^c^ *KIR2DL2/2DL3 – C1/C1: P =* 0.023, OR = 0.6 (0.4 – 0.9) to *per se vs*. controls.

**Figure 1:**
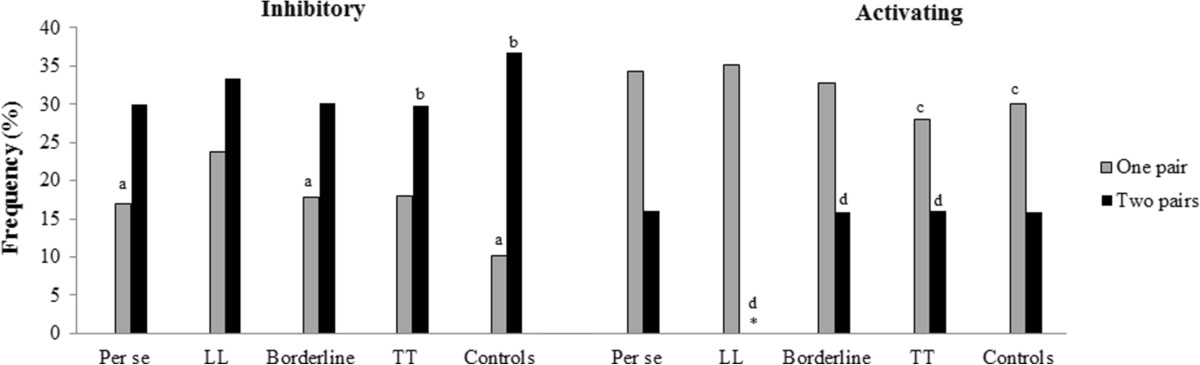
**Frequencies of pairs of ligands KIR-HLA Class I inhibitory and activating in leprosy patients and their clinical forms and healthy individuals in the population of Rondonopolis, MT. NOTE:** Bars diagram depicting the frequency of pairs of KIR-HLA inhibitory and activacting. TT: Tuberculoid, Borderline (BL, BB, BT), LL: Lepromatous. ^*^Represents that the LL group presented results of 0% for two activacting pairs. ^a^To one inhibitory pair: *P =* 0.006, OR = 1.8 (1.2 – 2.7) to *per se vs*. controls and *P* < 0.001, OR = 2.3 (1.5 – 3.6); to Borderline *vs*. controls. ^b^To two inhibitory pairs: *P =* 0.001, OR = 0.4 (0.2 – 0.8) to TT *vs*. controls. ^c^To one activating pair: *P =* 0.047, OR = 1.8 (1.0 – 3.1) to TT *vs*. controls. ^d^To two activating pairs: *P =* 0.024, OR = 11.0 (0.6 – 19.8) to TT *vs*. LL and *P =* 0.034, OR = 9.0 (0.5 – 15.1) to Borderline *vs*. LL.

We then investigated the influence of the number of KIR-HLA class I pairs, inhibitory and activating, on the development of leprosy and its clinical forms (Figure 1). There were significant differences between the Total Patient Group and Control Group (16.9% vs. 10.1%; *P-*value = 0.006; OR = 1.8; 95% CI = 1.2-2.7) and between the Borderline Group and the Control Group (17.8% vs. 10.1%; *P-*value < 0.001; OR = 2.3; 95% CI = 1.5-3.6) when only one pair of inhibitory ligands was present. When two pairs were present, there was a significance difference between the TT and Control Groups (28.3% vs. 36.7%; *P-*value = 0.001; OR = 0.4; 95% CI = 0.2-0.8). In the analysis of pairs of activating KIR-HLA class I ligands, when just one pair was present, there was a statistically significant difference between the TT and Control Groups (46.6% vs. 32.6%; *P-*value = 0.047; OR = 1.8; 95% CI = 1.0-3.1). When two pairs were present, there were statistically significant differences between the TT and LL Groups (20.0% vs. 0%; *P-*value = 0.024; OR = 11.0; 95% CI = 0.6-19.8), and Borderline and LL Groups (17.2% vs. 0%: *P-*value = 0.034; OR = 9.0; 95% CI = 0.5-15.1).

Additionally, multivariate logistic regression analysis was performed using the stepwise method [13] to investigate relationships between epidemiological variables (age, gender, and ethnic group) and genetic factors (KIR genes) in the development of leprosy and its clinical forms (Table 4). With respect to genetic factors, only significant results from the Chi-square analysis were included in the model. For gender, there was a significant difference between the groups: LL vs. Control, TT vs. Control, TT vs. Borderline and TT vs. LL. For the variable ethnicity, there was only a significant difference between the TT and Control and TT and Borderline groups (Table 4). The *KIR2DL1* gene was negatively associated in the comparisons between the groups: Total Patient vs. Control (β = -2.2; OR = 0.1) LL vs. Control (β = -2.8; OR = 0.06), TT vs. Control (β = -3.7; OR = 0.02) and TT vs. Borderline (β = -1.8; OR = 0.1). In the presence of the homozygous C2 ligand (*KIR2DL1* - *C2*/*C2*) there were positive associations between the groups: Total Patient vs. Control (β = 0.4; OR = 1.5), TT vs. Control (β = 1.6; OR = 5.0) and TT vs. Borderline (β = 1.9; OR = 6.9). The difference in the frequency of the *KIR2DL2-C1* gene was significant for TT vs. Borderline and TT vs. Control Groups. There were positive associations for *KIR2DL2/2DL2-C1* on comparing the LL and Control (β = 1.6; OR = 5.0) and TT and Control Groups (β = 1.1; OR = 3.2) and negative associations between the Borderline and LL Groups (β = -1.5; OR = 0.2). The pair of *KIR2DS2-C1* activating receptors was significant only between the Total Patient and Control Groups (*P-*value = 0.002).

**Table 4:** ***Stepwise*** **logistic regression analysis of factors associated with the development of leprosy and its clinical forms**

Variable response	Variable independent	Coefficient (β)	*P*	OR	-2Log
*per se vs.* controls	*KIR2DL1*	-2.227	0.001	0.10	1060.2
	*KIR2DL1 - C2/C2*	0.431	0.022	1.54	
	*KIR2DS2 - C1*	0.537	0.002	1.71	
LL *vs*. controls	Gender	-1.217	0.047	0.29	141.9
	*KIR2DL1*	-2.815	0.001	0.06	
	*KIR2DL2/2DL2 - C1*	1.612	0.042	5.01	
Borderline *vs*. controls	*KIR2DL1*	-1.868	0.001	0.15	936.9
TT *vs*. controls	Gender	0.755	0.017	2.13	288.5
	Ethnic	0.365	0.048	1.44	
	*KIR2DL1* ^e^	-3.708	0.001	0.02	
	*KIR2DL1 - C2/C2*	1.612	0.001	5.01	
	*KIR2DL2 - C1*	1.452	0.001	4.27	
	*KIR2DL2/2DL2 - C1*	1.173	0.059	3.23	
TT *vs*. Borderline	Gender^a^	0.947	0.002	2.57	281.7
	Ethnic	0.410	0.017	1.50	
	*KIR2DL1*	-1.809	0.001	0.16	
	*KIR2DL1 - C2/C2*	1.932	0.001	6.90	
	*KIR2DL2 - C1*	1.840	0.001	6.30	
TT *vs*. LL	Gender	1.783	0.004	5.95	82.4
Borderline *vs*. LL	*KIR2DL2/2DL2 - C1*	-1.579	0.024	0.206	151.3

**NOTE:** To this analysis, only KIR genes that were significant in the chi-square analysis were considered. (β): Coefficient of variables; -2Log: Logarithm of chance. Tuberculoid (TT), Borderline (BL, BB, BT), and Lepromatous (LL).

When only the HLA ligands of the KIR gene were evaluated, they were in equilibrium of Hardy-Weinberg, however no significant differences in their frequencies were found between groups.

## Discussion

Studying the impact of genes on infectious diseases such as leprosy in endemic regions is extremely important because it provides information about the contributions of host genetic factors and the environment in which the individual lives on the development of the disease. Historically in Brazil, the Mid-Western region has always had high rates of new cases of leprosy and the least favourable evolution of endemics in Brazil [14]. A recent study conducted by Magalhães et al. [15] assessed the pattern of leprosy in the State of Mato Grosso. The data revealed that the evolution of the disease was related to the process by which the region was settled, with successive migrations causing changes in the epidemiological structure due to the poor living conditions of the migrant population associated with other factors of infection in the disease process.

The current study shows that men are more affected by leprosy than women; the literature presents conflicting results regarding the prevalence of leprosy with respect to gender [16, 17]. Some authors claim that the greater social contact between men and their frequent exposure to risk environments can contribute to the increased number of cases [18]. Others claim that the prevalence of the disease in men is not universal and that, when related to leprosy reactions, women who are pregnancy or breastfeeding are at greater risk due to immunological and genetic characteristics [17]. In the evaluation of ethnic background, the highest frequency was found in Afro-Brazilians followed by Caucasians, which reflects the greater proportion of half-castes in the population of Mato Grosso.

**Figure 2:**
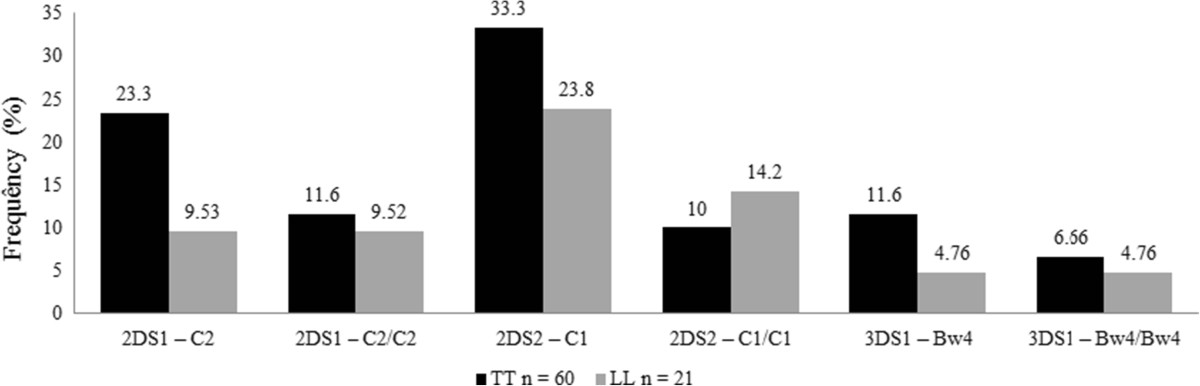
**Distribution of the frequencies of KIR genes activating in the presence of their ligands HLA Class I in patients with the clinical forms Tuberculoid and Lepromatous in the population of Rondonopolis, MT. NOTE:** Bars diagram depicting the frequency of KIR genes activacing genes and their respective ligands HLA Class I in patients with the Tuberculoid and Lepromatous clinical forms; the values in parentheses correspond to the percentages of each pair KIR-HLA. TT: Tuberculoid; LL: Lepromatous.

The analysis of the distribution of the KIR genes highlights differences in the frequencies of activating genes between the TT and LL forms of the disease, both separately and in the presence of the correlated HLA ligands. Figure 2 compares these two clinical forms with respect to activating genes and their ligands; there is a higher frequency of all KIR-HLA combinations in the TT compared to the LL form except for the *KIR2DS2-C1/C1* combination, thus supporting the results found by Franceschi et al. [10]. The results for activating genes (Table 2) are also in accordance with the results described by Franceschi et al. [10], who reported a higher frequency of the *KIR2DS1*, *2DS2*, *2DS3* and *2DS4* genes in TT compared to LL patients. The initial step in eliminating *M. leprae* requires the effective participation of the innate immune system, and thus NK cells play an important role. The activation of NK cells results in the production of IFN-γ, the main cytokine involved in the activation of macrophages, which promote the death of phagocytosed mycobacteria. Furthermore, the stimulation of macrophages by IFN-γ results in the production of TNF, which along with IFN-γ contributes to the activity of these cells against mycobacteria [19]. The higher frequency of activating genes in the group of TT patients compared to the LL group may enable a more efficient activation of NK cells, resulting in a low bacillary load and fewer skin and nerve lesions, i.e., a milder disease, thereby protecting patients against the most severe form of the disease.

In the analysis of activating KIR genes, the KIR2DS2-C1 pair was significantly more common in the Total Patient and TT Groups than the Control Group. It is important to note the presence of a strong binding imbalance between the KIR2DL2 and KIR2DS2 genes [9], as was confirmed in this study. The KIR2DL2/2DS2 haplotype frequencies were higher than expected in the Total Patient and Control Groups. Considering the presence of linkage disequilibrium between these two genes, the presence of the association may be attributable to the high frequency of the KIR2DL2 gene for both Groups.

The function of the KIR genes in the immune response is highly dependent on the HLA molecules expressed on the surface of target cells; the KIR-HLA interaction can result in either activation or inhibition of NK cells. The recognition of specific HLA molecules by inhibiting KIRs is well established [20], with a hierarchy of inhibition of the KIR2DL molecules: 2DL1-C2/C2 has the greatest inhibitory potential, followed by 2DL2-C1 and 2DL3-C1 [21]. In this study, was found a tendency of association for the gene KIR2DL1-C2C2 between the BL and BT forms, which are two opposite clinical forms, being BL nearer to the lepromatous pole. The results suggest that greater inhibition of NK cells may occur in these patients, leading to a more severe manifestation of the disease.

The distribution of the *KIR2DL3* and *KIR2DL2* inhibitory genes, and whether they were homozygous or heterozygous, was observed in this study, supporting the hypothesis that these genes segregate as alleles from a single locus [21, 22]. Interesting results were found for *KIR2DL2/2DL2-C1*; its frequency was higher in all patient groups compared to the Control Group, although the difference was only significant for the Total Patient, TT and LL Groups, suggesting that the inhibitory effect of *KIR2DL2/2DL2-C1* may contribute to the development of leprosy, but mainly contributes to a worse prognosis in *M. leprae* infections.

It is important to highlight the results observed in the analysis of the number of pairs of KIR-HLA ligands. The Total Patient Group had a more inhibitory profile due to the higher frequency of only one inhibitory pair than the Control Group, which may favour the development of the disease. A similar result was found for the Borderline Group of patients compared to the Control Group. Interestingly, in the analysis of the activating pairs of ligands, a higher frequency of two pairs was observed in the borderline patients compared to the LL Group. The interpolar forms, termed borderline, comprised the majority of the cases. The analysis of the KIR-HLA ligand pairs in this group showed a more inhibitory profile relative to the Control Group, added to a more activating profile compared to the LL Group. These individuals have a characteristic immune instability against the mycobacteria, which means that there is great variation in their clinical manifestations, whether of the skin, nerves, or systemic involvement. The balance between activation and inhibition of NK cells may explain the ill-defined characteristics observed in these patients.

The results of the multivariate analysis, in addition to confirming the influence of the KIR genes and their HLA ligands on the immunopathology of leprosy and its different forms, demonstrated the influence of gender. Interestingly, the LL Group presented a negative association with respect to gender compared to the Control Group. Thus, there were fewer females in the LL Group compared to the Control Group. On the other hand, there was a positive association with respect to gender on comparing the TT Group: TT vs. Control (β = 0.7; OR = 2.1), TT vs. Borderline (β = 0.9; OR = 2.5) or TT vs. LL (β = 1.7; OR = 5.9). Among the groups, the TT Group was the only one that comprised more women than men. These results suggest that being a woman is a protective factor against the most severe form and spread of the disease, in addition to contributing to its mildest manifestation with susceptibility to the TT form. Specific immune responses to autoimmune and infectious diseases differ between men and women. The female sex hormone, oestrogen, can increase the production of INF-γ and IL-2, thereby influencing the immune response in women. Gonadotropin-releasing hormone (GnRH) is involved in the maturation of the thymus and exerts a potent stimulatory effect, leading to increases in IL-2 and its receptor (IL-2R), INF-γ and activation of CD4^+^ helper T cells [23]. The male androgens, on the other hand, are related to the inhibition of T and B cell immune response and are found at low levels in men suffering from autoimmune diseases such as systemic lupus erythematosus [24].

With respect to ethnicity, the multivariate analysis demonstrated a positive association between TT and controls (β 0.3; OR 1.4) and between TT and Borderline (β 0.4; OR 1.5). For both groups, the TT Group had a higher frequency of Afro-Brazilians. It is possible that the migrations that occurred during settlement of the State of Mato Grosso influenced the ethnic composition of the population, whereby individuals classified as of mixed descent result from miscegenation between Afro-Brazilians and Indians.

The significant difference in the percentage of the *KIR2DL1* gene in the Total Patient and Borderline Groups compared to the Control Group in the Chi-square analysis was also significant in some multivariate analysis comparisons; however, in both cases the results were not correlated with the development of the disease or its different forms. *KIR2DL1-C2/C2*, which was not significant in the Chi-square analysis, was significantly different in the comparison between the Total Patient and Control groups; however, this did not result in associations with the disease or its clinical forms. The *KIR2DL2/2DL2-C1* inhibitory pair, which was significant in the Chi-square analysis, was also significant in the multivariate analysis (LL *vs.* Control; Borderline vs. LL), with a tendency being seen in the comparison between the TT *vs.* Control Groups. The significance of *KIR2DL2-C1* in the multivariate analysis may be due to the higher frequency of this pair in the TT Group (45.0%), but without implications for the pathogenesis of the disease. An equivalent result was observed for *KIR2DS2-C1*, whose significance, found when comparing the Total Patient and Control Groups, is probably due to a linking imbalance with *KIR2DL2* as reported for the two groups in this study.

## Conclusion

The current study shows that activating and inhibitory KIR genes in the presence of their corresponding HLA ligands may have some effect on the development of leprosy and its clinical forms, seen particularly with the strong presence of activating genes in the TT Group. The balance between activating and inhibitory genes may interfere with the progression of the disease to milder or more aggressive forms, or even cause it to vary between the two poles with indeterminate forms, thus highlighting the role of NK cells in the immunopathology of the disease.

The highlight of this study was identifying the profiles of patients with leprosy from a hyperendemic area of the country and comparing them to a group of extremely well matched healthy individuals.

## Authors’ original submitted files for images

Below are the links to the authors’ original submitted files for images. Authors’ original file for figure 1 Authors’ original file for figure 2
