# Supplementary figures and images for: Influence of KIR genes and their HLA ligands in the pathogenesis of leprosy in a hyperendemic population of Rondonópolis, Southern Brazil
Source: BMC Infect Dis. 2014 Aug 12;14:438. doi: 10.1186/1471-2334-14-438 (PMC4141108; doi:10.1186/1471-2334-14-438)

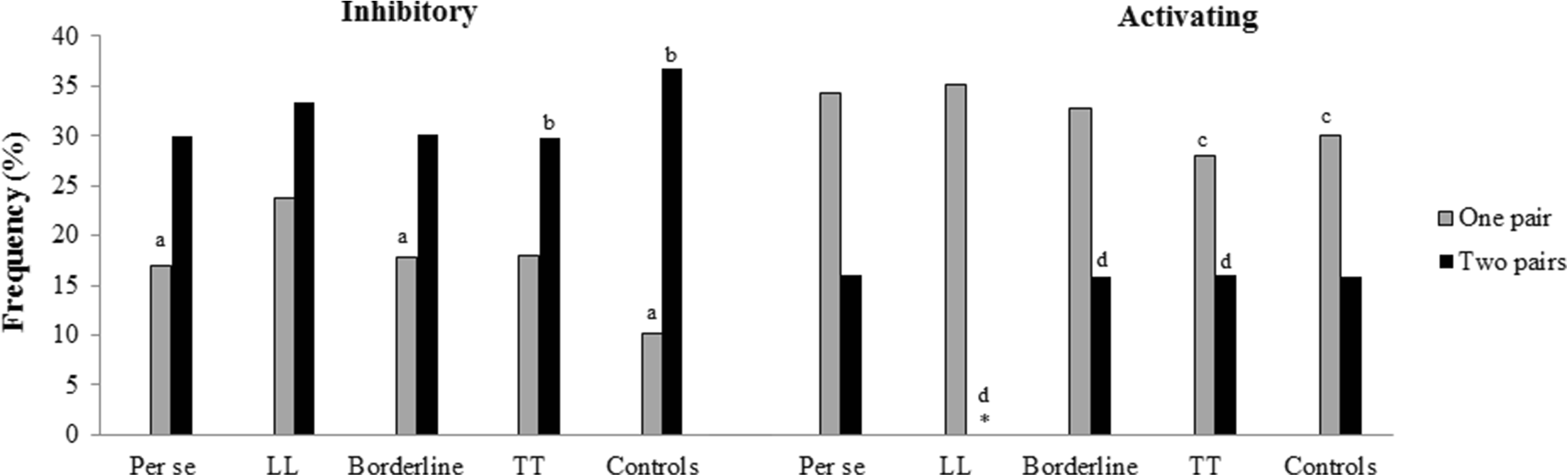

Supplement: Supplementary file 1 — Authors’ original file for figure 1 [file 12879_2013_3739_MOESM1_ESM.tif]

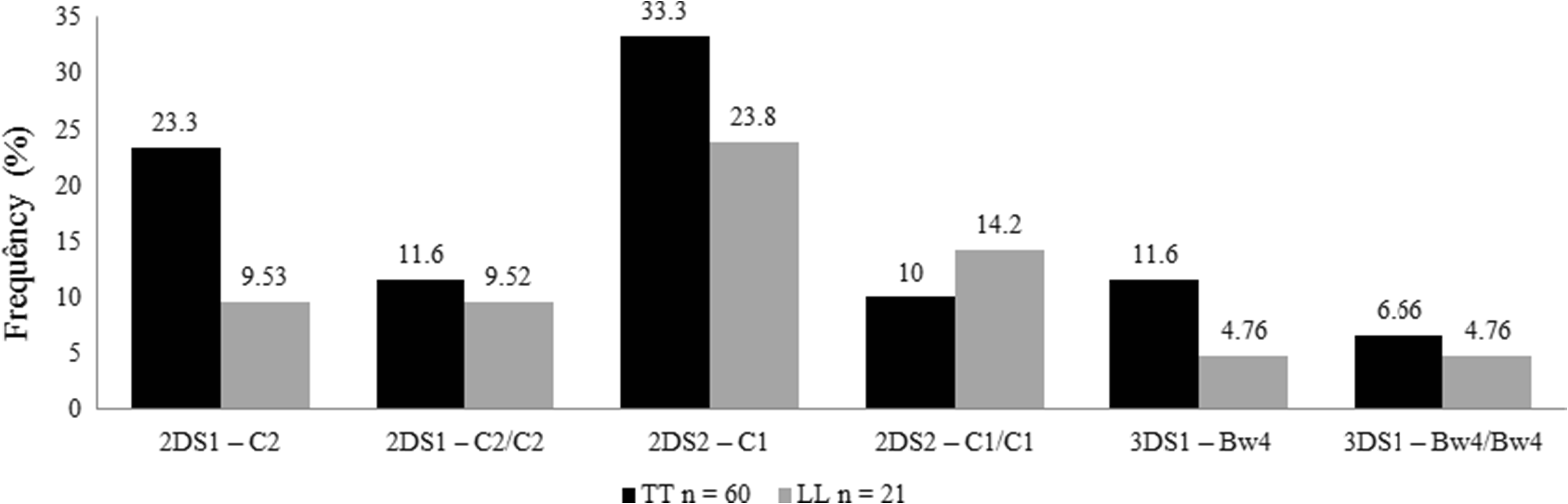

Supplement: Supplementary file 2 — Authors’ original file for figure 2 [file 12879_2013_3739_MOESM2_ESM.tif]
